# Comment on: Obesity is Associated with Improved Postoperative Overall Survival, Independent of Skeletal Muscle Mass in Lung Adenocarcinoma by Lee et al.

**DOI:** 10.1002/jcsm.13060

**Published:** 2022-08-14

**Authors:** Duk‐Hee Lee

**Affiliations:** ^1^ Department of Preventive Medicine, School of Medicine Kyungpook National University Daegu South Korea

I have read the article by Lee et al.^1^ with great interest. This article reported favourable post‐operative overall survival among obese patients with lung adenocarcinoma, a phenomenon known as the obesity paradox. Besides lung cancer, a better prognosis among overweight or obese patients has been repeatedly observed among patients with a broad range of diseases. For example, in 2021, this journal published several articles regarding the obesity paradox among patients with stroke,^2^ sepsis,^3^ and heart failure.^4^

Among several possible explanations for the obesity paradox, the methodological limitation of body mass index (BMI) as a marker of obesity has been considered a major underlying mechanism.^5^ BMI does not delineate adipose tissue distribution or distinguish between fat and lean body mass. Thus, body composition phenotypes have been considered as missing links in the obesity paradox.^6^

However, Lee et al.^1^ clearly demonstrated that a better prognosis among obese lung cancer patients was observed regardless of skeletal muscle mass, which was objectively assessed using computed tomography. They also observed that an increase in visceral adiposity was related to the improved overall survival despite the well‐known adverse effect of visceral obesity on cardiometabolic risk. These findings suggest the possibility of the real benefit of increased adiposity among patients with lung cancer.

Although largely unknown to researchers and clinicians, human adipose tissue plays a role as a storage organ for various lipophilic environmental contaminants. The most well‐known chemicals are compounds classified as persistent organic pollutants (POPs), which show strong lipophilicity, resistance to degradation, and long half‐lives of several years to decades.^7^ Examples of POPs are organochlorine pesticides, polychlorinated biphenyls (PCBs), dioxins, and polybrominated diphenyl ethers.^7^, ^8^ In addition to POPs, many less lipophilic chemicals with short half‐lives have been detected in human adipose tissue, including polyaromatic hydrocarbons, bisphenol A, phthalates, triclosans, and nonylphenols.^9^, ^10^ Therefore, human adipose tissue in modern society can be seen as a dump to accumulate exogenous chemicals that are not easily metabolized and excreted from the body.

The production and use of many individual chemicals belonging to POPs have been banned for several decades because of possible harm to wild animals and humans.^11^ However, humans are still living with continuous exposure to a complicated mixture of these chemicals, primarily due to the wide contamination of the food chain.^12^ Exposure to POPs begins at the fetal stage because POPs can be easily transported from the mother to the fetus.^13^ Infants are continuously exposed to POPs through breastfeeding after birth because breast milk contains high concentrations of POPs.^14^

Compared with the earlier period when POPs were actively used, the exposure dose of POPs in contemporary society is much lower. Recently, however, chronic exposure to low‐dose POPs has become a great concern to human health. For example, in epidemiological studies based on the general population with only background exposure to these chemicals, chronic exposure to low‐dose POPs has been linked to the increased risk of many diseases of the endocrine, immune, nervous, and reproductive systems.^15^, ^16^, ^17^, ^18^ Importantly, low‐dose POPs can act as mitochondrial toxins.^19^, ^20^

The toxico kinetics of POPs can explain puzzling findings that suggest the benefit of increased adiposity, such as the obesity paradox.^21^ Once lipophilic chemicals with long half‐lives, such as POPs, enter the human body from external exposure sources, they are primarily stored in adipose tissue and are very slowly released into the circulation for elimination.^22^ From the viewpoint of the whole‐body system, it can be seen as a protective mechanism because adipose tissue may be the safest place to store POPs. Therefore, if humans are exposed to the same levels of environmental POPs, having more adipose tissue can be beneficial because the storage of POPs in adipose tissue can reduce their burden on other critical organs.^21^

Several animal experiments have demonstrated the protective roles of adipose tissues against POPs. For example, diet‐induced obesity in rats increased survival time after exposure to a lethal dose of 2,3,7,8‐tetrachlorodibenzo‐p‐dioxin, the most toxic compound among POPs.^23^ Moreover, treatment with PCBs induced impaired glucose homoeostasis only in lean mice, but not in obese mice; however, weight loss in obese mice developed glucose impairment.^24^

In addition, POPs in adipose tissue can explain the worse prognosis of lung cancer or other cancer patients who experience unintentional weight loss during pre‐diagnosis, peri‐diagnosis, and post‐diagnosis.^25^, ^26^, ^27^ Currently, there are several explanations for this phenomenon, such as poor tolerance to treatment, high severity, decreased performance status, or co‐morbid conditions. However, POPs are released from adipose tissue into circulation during weight loss due to adipose tissue shrinkage, and the amount of POPs released is proportional to the magnitude of weight loss.^28^ Therefore, the dynamics of POPs may be a possible mechanism linking unintentional weight loss and poor prognosis in cancer patients.

Furthermore, even intentional weight loss can have an unexpected drawback, although intentional weight loss through lifestyle modification is generally recommended to overweight or obese individuals. For example, the Action for Health in Diabetes (Look AHEAD) study, a large randomized controlled trial that investigated the effects of intensive intentional weight loss in obese patients with Type 2 diabetes, did not report any long‐term benefits of intentional weight loss on cardiovascular disease or cognition despite many short‐term benefits.^29^, ^30^ This unanticipated consequence can be attributed to the POP dynamics observed during intentional weight loss.^31^, ^32^ It is important to note that weight fluctuation, a common result of intentional weight loss, can lead to redistribution of POPs from adipose tissue to critical organs, as observed in animal experimental studies.^33^

The role of adipose tissue in storing POPs and other environmental pollutants may not be trivial and can explain many puzzling findings on obesity observed in epidemiological studies. In contrast to the prevailing idea of obesity, adipose tissue may be the first line of defence against the possible harmful effects of lipophilic chemical mixtures. If this role of adipose tissue becomes more significant in patients with cancer or other chronic diseases, the obesity paradox can be explained. Investigation of the interrelationship between adipose tissue and lipophilic chemical mixtures is urgently required.

## Funding

This work was supported by the National Research Foundation (grant number 2019R1A2C1008958) and funded by the Ministry of Science and ICT of the Republic of Korea.
